# Congenital tracheal diseases: diagnosis and management

**DOI:** 10.1186/1749-8090-10-S1-A84

**Published:** 2015-12-16

**Authors:** Vu Huu Vinh

**Affiliations:** 1 Department of Thoracic Surgery, Choray Hospital, Hochiminh City, Vietnam

## Background/Introduction

Congenital tracheal diseases are less common than congenital cardiac.

## Aims/Objectives

They contain two lesions: anomaly and stenosis. They may associate with other congenital lesions especially pulmonary artery sling. Tracheal stenosis is the main factor causing airway obstruction. Depend on the degree of tracheal stenosis, clinical manifestation ranges from having some episodes of stridor to severe respiratory failure and death. Reconstruction surgery is the method of choice for severe cases.

## Method

Retrospective review congenital tracheal disease cases that underwent reconstruction surgery for over two years (from Aug. 2013 to Feb. 2015). Tracheal stenosis parts were managed using slide tracheoplasty technique. As for other congenital lesions, only pulmonary sling was reconstructed, together with tracheoplasty.

## Results

16 patients (male 7; female 9) were operated. Ages range from 3 - 12 months (average: 7.9). Other congenital lesions include: sling: 12/16; ring: 1/16; VSD: 2/16; no anus: 2/16. Result is excellent in 14 cases: good in one. Mortality: 1.

## Discussion/Conclusion

Reconstruction surgery is feasible for congenital tracheal diseases. Result is excellent. Slide tracheoplasty should be applied in all cases to manage tracheal stenosis parts. Segments resection is not recommended for any part of the trachea.

